# Conjugated bile acids are elevated in severe calcific aortic valve stenosis

**DOI:** 10.1016/j.jlr.2025.100830

**Published:** 2025-05-22

**Authors:** Hannah Zhang, Negar Atefi, Arun Surendran, Jun Han, David R. Goodlett, Davinder S. Jassal, Ashish Shah, Amir Ravandi

**Affiliations:** 1 Cardiovascular Lipidomics Laboratory, St. Boniface Hospital, Albrechtsen Research Centre, Winnipeg, MB, Canada; 2 Department of Physiology and Pathophysiology, Rady Faculty of Health Sciences, University of Manitoba, Winnipeg, MB, Canada; 3 Mass Spectrometry & Proteomics Core Facility, Rajiv Gandhi Centre for Biotechnology, Thiruvananthapuram, Kerala, India; 4 Genome British Columbia Proteomics Centre, University of Victoria, Victoria, BC, Canada; 5 Division of Medical Sciences, University of Victoria, Victoria, BC, Canada; 6 Department of Biology and Microbiology, University of Victoria, Victoria, BC, Canada; 7 Section of Cardiology, Department of Internal Medicine, Rady Faculty of Health Sciences, University of Manitoba, Winnipeg, MB, Canada; 8 Precision Cardiovascular Medicine Group, St. Boniface Hospital Research, Winnipeg, MB, Canada

**Keywords:** aortic valve calcification, bile acids, calcific aortic valve stenosis, conjugated bile acids

## Abstract

Calcific aortic valve (AV) stenosis (CAVS) is a disease associated with significant morbidity and mortality in the aging population. Recently, bile acids have been shown to play a significant role in many disease processes, and untargeted metabolomic analyses of CAVS patient valves have shown a disrupted bile acid pathway. We aimed to understand the changes in human valvular bile acids in relation to CAVS severity. A total of 100 human AVs were collected from patients undergoing AV replacement surgery. Bile acids were quantified by ultrahigh performance liquid chromatography coupled to MS/MS. Patients with mild aortic stenosis (AS) showed a distinct valvular bile acid composition compared with moderate and severe AS groups, with five bile acids being significantly elevated in patients with moderate and severe AS. These included norcholic, nordeoxycholic, glycodeoxycholic, glycocholic, and taurodeoxycholic acid. When classified by calcification score, five species were significantly different between mild and severe AS groups; four bile acids were similar when stratified based on AS severity. Using K-means clustering, we were able to distinguish valve severity by their bile acid composition. Grouping bile acids by conjugation and by primary versus secondary revealed that conjugated primary and secondary bile acids were significantly increased in stenotic valves compared with the mild AS group. Conjugated bile acids are significantly elevated in the valvular tissue of patients with severe calcific AS. These findings suggest a potential link between liver and gut microbiome physiology and bile acid pathways in contributing to the pathophysiology of valvular stenosis.

Calcific aortic valve (AV) stenosis (CAVS) is a prevalent, progressive valvular disease that poses significant clinical morbidity and mortality, especially in the aging population (1, 2). The disease spectrum ranges from aortic sclerosis—characterized by leaflet thickening and calcification without obstruction—to calcific aortic stenosis (AS), where leaflet stiffening leads to obstructive outflow disease. Aortic sclerosis affects approximately 21–26% of adults over the age of 65, whereas AS occurs in 2–9% of this population. Without treatment, symptomatic CAVS carries a poor prognosis (2, 3).

The pathogenesis of CAVS is complex and not fully understood. Once regarded as a passive degenerative process, emerging evidence now suggests that CAVS is driven by active cellular mechanisms. Early histopathologic studies reveal atherogenic lipoprotein accumulation, inflammatory cell infiltration, and microscopic calcification within the valve leaflets (3, 4, 5, 6, 7). As the disease progresses, leaflet thickening and stiffness lead to narrowing of the aortic orifice, increasing left ventricular pressure and potentially resulting in left ventricular hypertrophy and heart failure (8). Late-stage calcification is often irreversible, necessitating surgical or transcatheter valve replacement.

CAVS is typically classified into mild, moderate, and severe stages. Patients with early, mild disease are often asymptomatic and may remain undiagnosed until the disease advances. Understanding early disease progression is therefore critical to preventing irreversible valvular calcification and related complications.

Recent untargeted metabolomic analyses of AV tissues suggested a disruption in local bile acid pathways with increasing CAVS severity; however, the pathophysiology is still unclear (9). Although bile acids are traditionally linked to digestion, they are bioactive molecules involved in various physiological systems, including lipid metabolism, inflammation, and calcium-phosphate homeostasis, through the activation of receptors such as G-protein coupled receptors and farsenoid X receptors (FXRs) (10). They have even recently been shown to have a role in calcium-phosphate metabolism (11). Given their emerging role in calcific processes, this study aims to further investigate the specific changes in tissue and plasma bile acid profiles across varying severities of CAVS.

## Materials and Methods

### Chemicals

LC-MS grade water, acetonitrile, methanol, 2-propanol, formic acid, ammonium acetate, and ammonium formate were purchased from Thermo Fisher Scientific (Mississauga, ON). Chloroform was purchased from MilliporeSigma (Oakville, ON). Acetic acid and leucine enkephalin were purchased from Sigma-Aldrich (St. Louis, MO).

### Tissue collection, valve phenotyping, and patient grouping

AV leaflets were obtained from a total of 100 patients undergoing AV replacement surgery at St. Boniface Hospital (MB, Canada) between June 2014 and July 2015, with blood samples collected from a subcohort of 18 of these patients. The study was approved by the University of Manitoba Research Ethics Board and the St. Boniface Hospital Research Review Committee (B2014:034 and RRC/2014/1390, respectively). This study abides by the Declaration of Helsinki principles. Patient inclusion and exclusion criteria and sample collection have been previously described by Surendran *et al.* (9). The AV phenotype, measurements obtained by Doppler echocardiography and subsequent patient grouping, have also been previously described (9, 12). In brief, patients were grouped into three disease stages (mild, moderate, and severe) by two different metrics: mean pressure gradient (MPG) and calcification score. For MPG stratification, mild AS was defined by an MPG <20 mm Hg, moderate AS was defined as 20 mm Hg ≤MPG <40 mm Hg, and severe AS by an MPG ≥40 mm Hg. The degree of AV calcification was scored according to the previously described C-score (13, 14). A C-score value from 1 to 5 was assigned by a single operator for the ultrasound still frames to the whole valve. Nonthickened and noncalcified valves were classified as having score “1,” thickened but noncalcified valves as “2,” calcification spots covering less than one-third of the leaflet area as “3,” calcification spots covering less than two-thirds of the leaflet area as “4,” and calcification spots covering more than two-thirds of the leaflet area as “5.” Patients with a C-score of “1” or “2” were part of the mild AS group, a score of “3” was considered moderate AS, and scores of “4” or “5” were classified as severe AS. Healthy control plasma was from a previously published cohort (15).

### Extraction procedures and sample preparations

Frozen valve tissues were thawed on ice and cut into smaller fragments and weighed. Tyrode’s solution was added to the tissue samples to make a final concentration of 175 mg of raw tissue per ml. The samples were homogenized in bead ruptor tubes using an Omni Bead Ruptor 24. The final homogenates were transferred into microcentrifuge tubes and stored at −80°C until subsequent extraction. The blood samples were collected in EDTA-treated tubes and immediately centrifuged at 2,500 *g* for 10 min at 4°C to isolate plasma. Average time of blood collection to plasma separation and aliquoting was less than 30 min.

### Bile acid LC-MS/MS

Bile acids were quantitatively measured using an ultrahigh performance liquid chromatography (UPLC)-MS/MS method described previously with the use of standard substances for an expanded panel of 79 bile acids (16, 17). The information of the 79 bile acids is provided in supplemental Table S3. Tissue homogenates were thawed on ice and vortexed for 1 min at 3,000 rpm on a digital vortex-mixer. At room temperature, 100 μl of each homogenate was transferred to a 1.5-ml Eppendorf tube. Internal standard solution (50 μl) containing 14 deuterium-labeled bile acids (16) dissolved in 50% acetonitrile and 200 μl of acetonitrile were added to each tube. The concentrations of the 14 deuterium-labeled internal standards were 75 nM for the glycine-conjugated and taurine-conjugated bile acids and 25 nM for the unconjugated bile acids. The samples were vortexed for 1 min and then sonicated in an ice-water bath for 5 min, followed by centrifugal clarification at 21,000 *g* for 15 min at 10°C in an Eppendorf 5224R centrifuge. The supernatant of each sample was transferred to a 1-ml microvial and dried at 30°C under a flow of nitrogen gas inside an evaporator. The dried residue was reconstituted in 50 μl of 50% acetonitrile. To make calibration solutions, the mixed solution containing standard substances of all the measured bile acids (supplemental Table S3) was prepared with the same internal standard solution as what was added to each sample. This standard-substance solution was diluted in a ratio of 1–4 (v/v) with the internal standard solution to have 10 serially diluted calibration solutions. The concentrations of each bile acid in the calibration solutions ranged from 0.0001 to 10 μM. Aliquots of the resultant sample (15 μl) and calibration solutions were injected for the measurement of bile acids by UPLC-MS/MS in the multiple-reaction monitoring scanning mode. The UPLC-MS/MS instrument was an Agilent 1290 UPLC system coupled to a Sciex 4000 QTRAP mass spectrometer, which was operated with negative-ion electrospray ionization. A reversed-phase C18 column (2.1 ∗ 150 mm, 1.7 μm) was used for chromatographic separation, with the use of 0.01% formic acid in water and 0.01% formic acid in acetonitrile as the binary-solvent mobile phase for gradient elution at 55°C and 0.28 ml/min. LC-MS/MS data were acquired using the Sciex Analyst 1.7 software suite and processed using the Sciex MultiQuant 2.0 software. For data processing, linearly regressed, internal-standard calibration curves of individual bile acids were constructed with the data acquired from the calibration solutions within an appropriate concentration range for each compound. Concentrations of bile acids detected in the tissue samples were calculated by interpolating the calibration curves with the analyte-to-internal standard peak area ratios measured from the sample solutions. For those bile acids without their deuterium-labeled analogs available, deoxycholic acid-d4, glycodeoxycholic acid-d4, and taurodeoxycholic acid-d4 were used as the common internal standards for the unconjugated, glycine-conjugated, and taurine-conjugated bile acids, respectively, during the concentration calculation.

For quantitation of bile acids in human plasma, 30 μl of each thawed plasma sample was mixed with 90 μl of the internal standard solution and 90 μl of acetonitrile in a 1.5-ml Eppendorf tube. After vortex mixing for 30 s at 3,000 rpm and subsequent ultrasonication in an ice-water bath for 5 min, the samples were centrifuged at 21,000 *g* and 10°C for 15 min. The clear supernatants were taken out and mixed with 2 ml of water. The mixtures were subjected to cleanup by solid phase extraction on 100 mg/3 ml C18 cartridges. After sample loading and subsequent washing with 3 ml of water, bile acids bound on the C18 resin were eluted with 3 ml of acetonitrile under a 5-psi positive pressure. The collected effluents were dried at 30°C in a nitrogen evaporator. The dried residues were reconstituted in 90 μl of 50% acetonitrile. The resultant sample solutions were subjected to the same UPLC-MS/MS analysis as did for the tissue sample solutions.

### Statistical analysis

All statistical tests were performed with Python and GraphPad Prism. Data were log10 transformed for normalization. Values of 0 were replaced with 10^-6^ for analyses. Chi-square, one-way ANOVA, and *t*-tests were used to calculate the bile acid and clinical differences between the different severity groups. Pearson correlation was used to determine the relationships between bile acids and clinical factors, and linear regression was used to find the predictive value of significant bile acids. *P* values of under 0.05 were deemed significant and were corrected for multiple comparisons with Benjamini-Hochberg. Statistical outliers were determined with Grubb’s test and replaced with the mean values after outlier removal. K-means clustering was performed in Python by first performing principal component analysis to reduce the dimensionality of the normalized data, and the ideal number of clusters was determined using the Silhouette score and Calinski-Harabasz score.

## Results

### Patient demographics

Baseline parameters for the 100 patients are outlined in Table 1. A total of 69 males and 31 females were included in the study, with an overall average age of 69 ± 11.2 (range, 33–89 years). The average BMI was 29.3 ± 5.6 kg/m^2^. A total of 75% of patients had a history of hypertension with 66% of patients receiving antihypertensive treatment, and 42% had no smoking history. Twenty-nine patients had bicuspid AVs. When separated by MPG groups, defined by mild (MPG <20 mm Hg, n = 12), moderate (20 mm Hg ≤MPG <40 mm Hg, n = 39), and severe (MPG ≥40 mm Hg, n = 49), clinical parameters, such as age, sex, BMI, hypertension, and laboratory values were not different between the groups (Table 1). Smoking history and all measured echocardiography parameters were significantly different between the MPG groups except left ventricular ejection fraction.

**Table 1:** Baseline characteristics of the patient population, stratified by MPG

Parameter	Total (n = 100)	Mild (n = 12)	Moderate (n = 39)	Severe (n = 49)	*P*
Age (y)	69.2 ± 11.2	69.9 ± 10.3	71.2 ± (11.3)	67.4 ± (11.3)	0.293
Male, n (%)	69	9 (75)	25 (64)	35 (71)	0.68
Body surface area (m^2^)	1.97 ± 0.22	1.90 ± 0.25	1.97 ± 0.21	2.00 ± 0.19	0.306
BMI (kg/m^2^)	29.3 ± 5.6	26.24 ± 4.5	29.39 ± 5.0	30.60 ± 5.7	**0.041**
Hypertension, n (%)	75	8 (67)	30 (77)	37 (76)	0.77
Smoking history, n (%)					
Current	10	0 (0)	5 (13)	5 (10)	
Previous	48	6 (50)	25 (64)	17 (35)	**0.03**
Never	42	6 (50)	9 (23)	27 (55)	
Medications, n (%)					
Antihypertensive	66	7 (58)	26 (67)	33 (67)	0.83
Angiotensin-converting enzyme inhibitors	30	5 (42)	11 (28)	14 (29)	0.64
Acetylsalicylic acid	28	2 (17)	9 (23)	17 (35)	0.31
Angiotensin II receptor blockers	11	2 (17)	4 (10)	5 (10)	0.8
Statins	61	5 (42)	23 (59)	33 (67)	0.25
Laboratory					
LDL (mmol/l)	2.63 ± 1.02	3.03 ± 0.98	2.68 ± 1.00	2.49 ± 1.03	0.235
HDL (mmol/l)	1.36 ± 0.46	1.51 ± 0.50	1.27 ± 0.26	1.40 ± 0.56	0.198
Triglyceride (nmol/l)	1.49 ± 0.82	1.39 ± 0.80	1.44 ± 0.64	1.54 ± 0.96	0.788
Random glucose (mmol/l)	7.20 ± 2.73	6.34 ± 1.43	7.54 ± 2.88	7.14 ± 2.83	0.404
Creatinine (μmol/l)	90.48 ± 38.07	98.73 ± 76.71	88.49 ± 32.18	90.04 ± 28.30	0.717
Echocardiogram data					
Bicuspid valve, n	29	5 (42%)	10 (26%)	14 (29%)	0.56
Calcification score, n (%)					
1	8	6 (50)	0 (0)	2 (4)	
2	27	2 (17)	15 (38)	10 (20)	
3	40	3 (25)	17 (46)	20 (41)	**<0.001**
4	18	0 (0)	6 (15)	12 (24)	
5	7	1 (8)	1 (3)	5 (10)	
Peak aortic jet velocity (m/s)	4.03 ± 0.95	2.51 ± 0.79	3.74 ± 0.28	4.64 ± 0.78	**<0.001**
Peak transvalvular gradient (mm Hg)	68.8 ± 30.5	22.79 ± 10.13	55.16 ± 8.66	90.86 ± 25.61	**<0.001**
Mean transvalvular gradient (mm Hg)	41.6 ± 18.8	11.83 ± 4.45	32.86 ± 4.92	55.84 ± 14.57	**<0.001**
Aortic valve area (cm^2^)	0.95 ± 0.40	1.56 ± 0.71	0.93 ± 0.26	0.82 ± 0.23	**<0.001**
Indexed aortic valve area (cm^2^/m^2^)	0.51 ± 0.35	0.81 ± 0.37	0.48 ± 0.13	0.47 ± 0.43	**0.00801**
Left ventricular mass index (g/m^2^)	127.4 ± 33.0	148.3 ± 36.1	116.6 ± 24.6	130.8 ± 35.4	**0.0075**
Left ventricular ejection fraction (%)	56.4 ± 9.4	55.4 ± 7.2	55.6 ± 10.9	57.5 ± 8.6	0.588

Groups (mild: MPG <20 mm Hg; moderate: 20 mm Hg ≤MPG <40 mm Hg; and severe: MPG ≥40 mm Hg). *P* values were obtained after subgroup comparison, with *P* values <0.05 bolded. Values are mean ± SD, mean (range), numbers or % as applicable. Chi-square test was used for categorical variables, and one-way ANOVA (post hoc Tukey test) was used for continuous variables to assess for statistical significance between sample groups.

### Bile acid composition of human AVs

**Figure 1:**
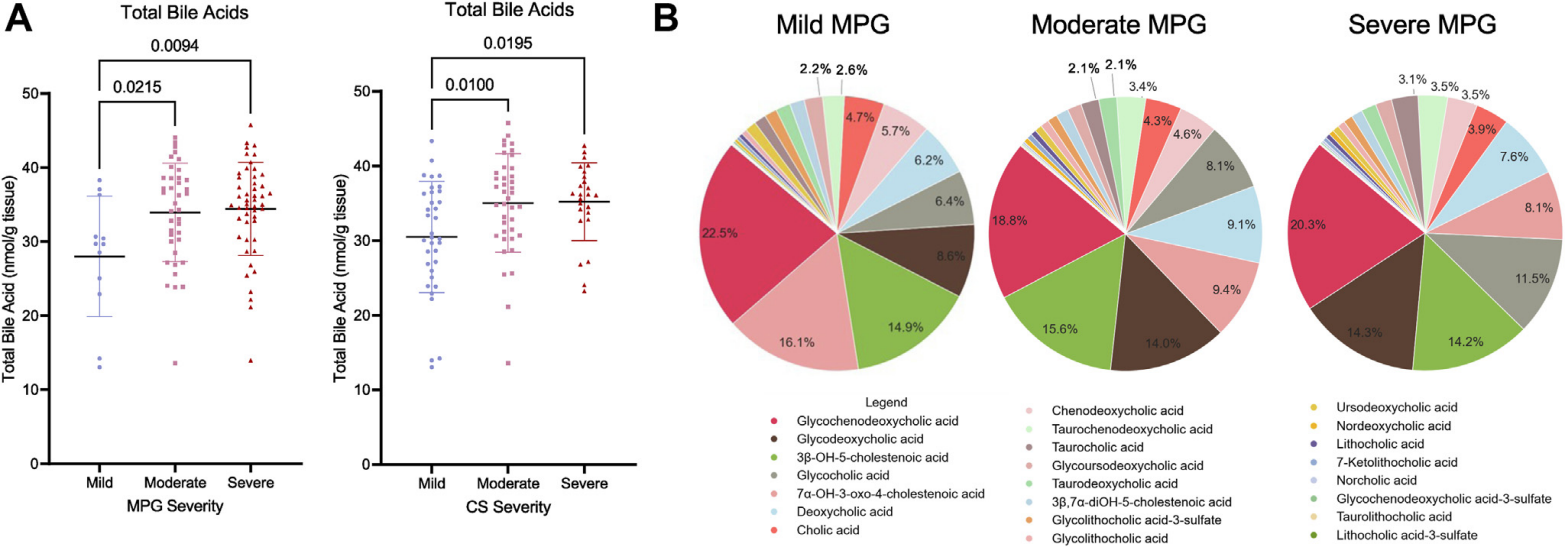
Bile acid composition of aortic valves (A) total bile acids in human aortic valve separated based on MPG and calcification score. B: Bile acid composition of aortic valves by severity group based on MPG (n = 100).

Group sizes are indicated in Table 1. When we separated the valvular tissues based on MPG, there was a significant increase in total valvular bile acids in moderate AS valves compared with mild AS (Fig. 1A). A similar increase was observed when valves were separated based on severity of calcification (Fig. 1A). When analyzing the bile acid composition of mild AS valves as defined by MPG, glycochenodeoxycholic acid is the most abundant, followed by 7α-OH-3-oxo-4-cholestenoic acid, 3β-OH-5-cholestenoic acid, glycodeoxycholic acid, glycocholic acid, and deoxycholic acid (Fig. 1B). In moderate and severe AS, glycochenodeoxycholic acid remained the most abundant bile acid in valvular tissue. Interestingly, glycocholic acid and glycodeoxycholic acid increased in relative abundance with increasing valvular severity, while the opposite was true of 7α-OH-3-oxo-4-cholestenoic acid.

### Valvular bile acid and MPG

**Figure 2:**
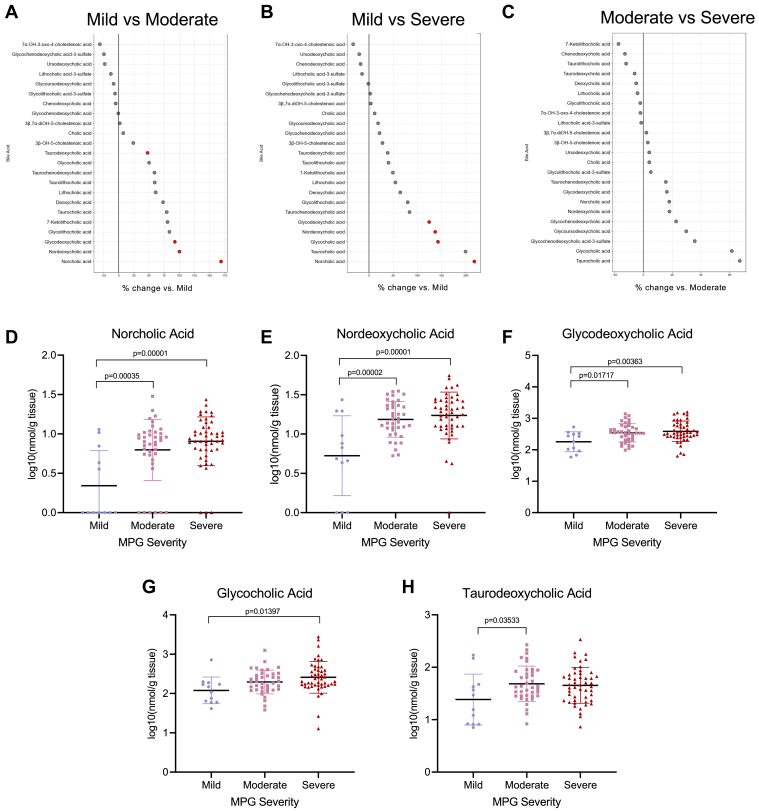
Valvular bile acid differences by MPG group. A–C: Forest plots of tissue bile acids comparing each of the severity groups. Red dots indicating statistically significant bile acids calculated by one-way ANOVA and with Benjamini-Hochberg correction. D–H: Norcholic, nordeoxycholic, glycodeoxycholic, glycocholic, and taurodeoxycholic acids are significantly different between severity groups when analyzed by one-way ANOVA with Benjamini-Hochberg multiple comparison adjustment. Significant *P* values <0.05 are as indicated on the graphs, and bars indicate SEM.

There were significant differences in bile acid composition of valvular tissue between MPG-based severity groups. Four bile acids were significantly elevated in the moderate compared with mild AS groups, and four were elevated in the severe compared with mild AS group (Fig. 2A, B). Specifically, tissue levels of norcholic acid, nordeoxycholic acid, and glycodeoxycholic acid differed significantly between the mild and severe AS groups, and the mild and moderate groups, with no significant difference between the moderate and severe AS groups (Fig. 2D–F). Glycocholic acid was significantly different between the mild and severe AS groups, whereas taurodeoxycholic acid differed between the mild and moderate AS groups (Fig. 2G, H). However, no significant differences in bile acids between moderate and severe AS patients were noticed when stratified by MPG (Fig. 2C).

### Valvular bile acid and calcification score

**Figure 3:**
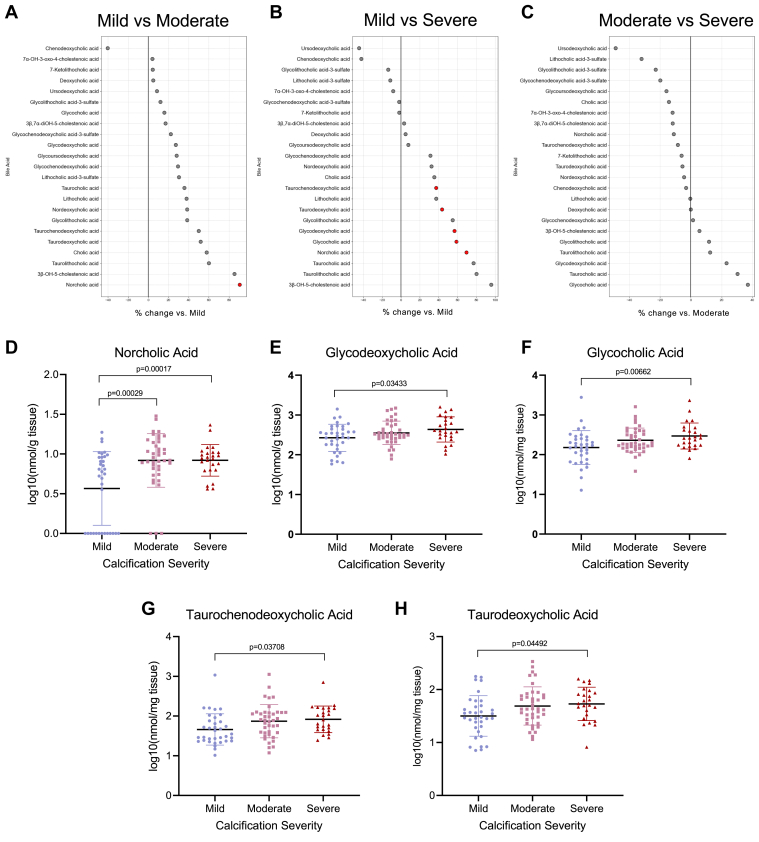
Valvular bile acid differences by calcification score. A–C: Forest plots of tissue bile acids comparing each of the severity groups. Red dots indicating statistically significant bile acids calculated by one-way ANOVA and with Benjamini-Hochberg correction. D–H: Norcholic, glycodeoxycholic, glycocholic, taurochenodeoxycholic, and taurodeoxycholic acids are significantly different between severity groups when analyzed by one-way ANOVA with Benjamini-Hochberg multiple comparison adjustment. Significant *P* values <0.05 are as indicated on the graphs, and bars indicate standard error.

Patients were grouped based on their calcification scores into the following categories: mild (calcification score 1–2, n = 35), moderate (calcification score 3, n = 40), and severe (calcification score 4–5, n = 25) AS. After adjusting for multiple comparisons, five bile acids in tissue samples showed significant differences among these severity groups, one of which was different between mild and moderate AS groups, and five of which were different between mild and severe AS groups (Fig. 3A-B). Norcholic acid levels differed significantly between mild and moderate as well as mild and severe valves (Fig. 3D). Glycodeoxycholic acid, glycocholic acid, taurochenodeoxycholic acid, and taurodeoxycholic acid levels were significantly different only between mild and severe AS groups (Fig. 3E–H). Of note, four bile acids were significantly different in both MPG and calcification score stratifications: norcholic, glycodeoxycholic, glycocholic, and taurodeoxycholic acids. Consistent with the MPG classification analysis, no bile acids were significantly different between moderate and severe valves based on calcification score (Fig. 3C).

**Figure 4:**
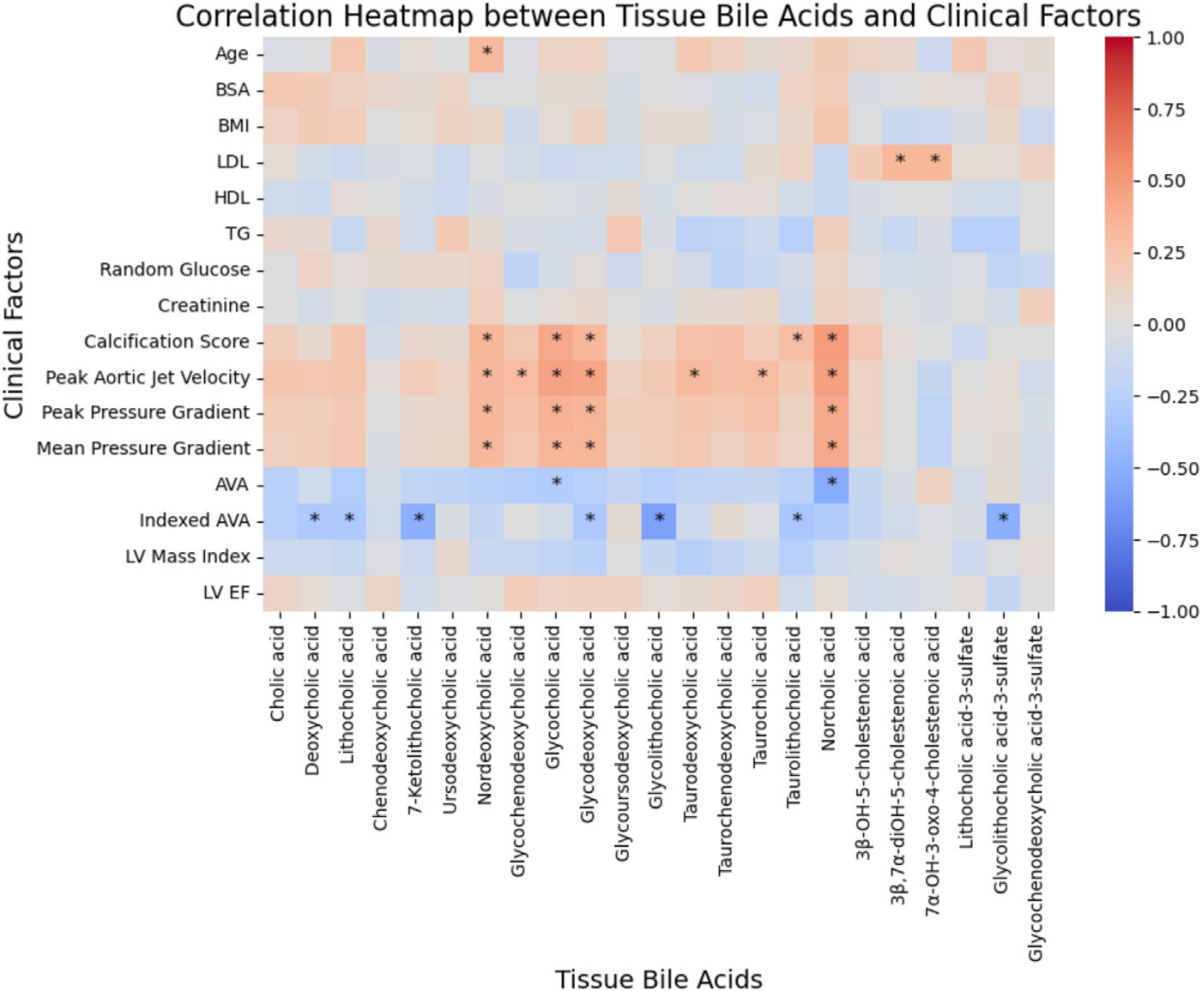
Pearson correlation matrix of the relationships between tissue bile acids and clinical factors. All *P* values were adjusted for multiple comparisons, and “∗” indicates statistical significance (*P* < 0.05) after adjustment.

Several valvular tissue bile acids were significantly correlated with clinical echocardiographic measurements, whereas only nordeoxycholic acid was correlated with age, and no bile acids were correlated with body surface area, BMI, HDL, triglycerides, or random glucose or creatinine (Fig. 4). Two bile acids were significantly correlated with LDL levels, including 7α-OH-3-oxo-4-cholestenoic acid and 3β-OH-5-cholestenoic acid. Notably, similar bile acids were correlated with multiple echocardiographic parameters, including calcification score, peak aortic jet velocity, peak and MPGs, and AV area. The bile acids that had several significant correlations to echocardiographic data included nordeoxycholic acid, glycocholic acid, glycodeoxycholic acid, and norcholic acid (Fig. 4).

### Grouping by valvular tissue bile acids

**Figure 5:**
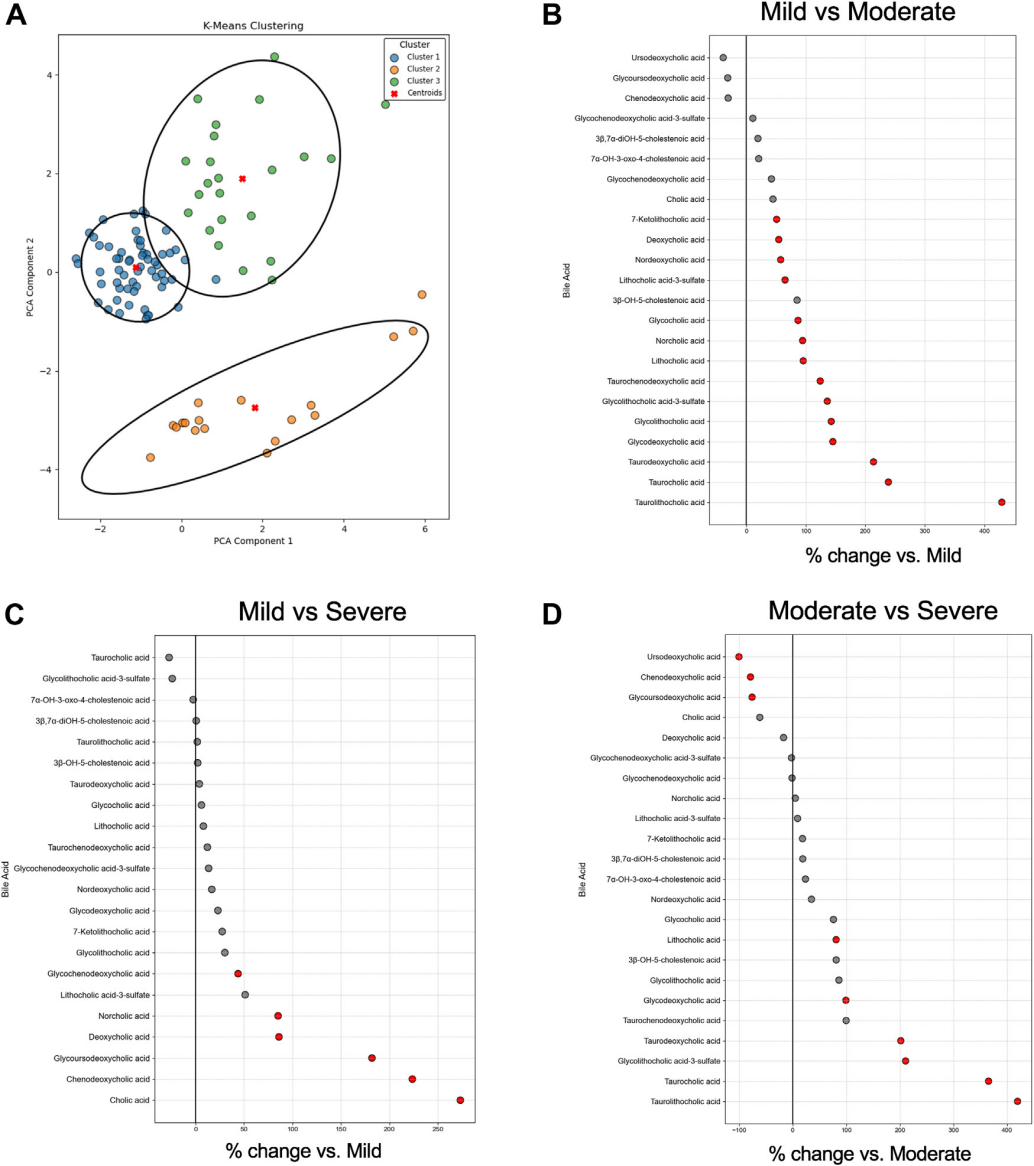
K-means clustering analysis of aortic valvular tissue bile acids. A: The principal component analysis plot of the K-means clusters. B–D: Forest plot of the significantly different bile acids between the different clusters, with cluster 1 being “severe,” cluster 2 as “moderate,” and cluster 3 as “mild.” Significant *P* values were deemed as *P* values <0.05 calculated by one-way ANOVA and Benjamini-Hochberg multiple comparison adjustment.

To determine if tissue bile acid profiles alone can separate patients based on valvular disease parameters, we performed K-means clustering, a machine learning algorithm, which separated our cohort into three distinct groups (Fig. 5A). When analyzing baseline clinical parameters of these groups, no clinical factors, such as age, sex, or BMI, were significantly different between them (supplemental Table S1). However, this unsupervised grouping separated our cohort along several echocardiographic parameters, with cluster 1 as patients with severe valvular disease, cluster 2 with moderate disease, and cluster 3 with mild disease. One-way ANOVA was performed to examine the differences in valvular bile acids between the clusters. Nine bile acids were significantly different between mild and moderate cluster valves (Fig. 5B). When comparing mild and severe clusters, 14 bile acids were significantly higher in severe compared with mild valves (Fig. 5C). Importantly, these 14 bile acids included all the bile acids identified as significant with MPG and CS analysis. Seven bile acids were significantly different between moderate and severe clusters, three of which were distinct from the bile acid changes seen in mild to severe valves, including cholic acid, chenodeoxycholic acid, and glycoursodeoxycholic acid (Fig. 5D). Therefore, using valvular bile acid profiles alone, patients can be separated into distinct severity groups.

### Conjugated secondary bile acids are higher in patients with severe disease

**Figure 6:**
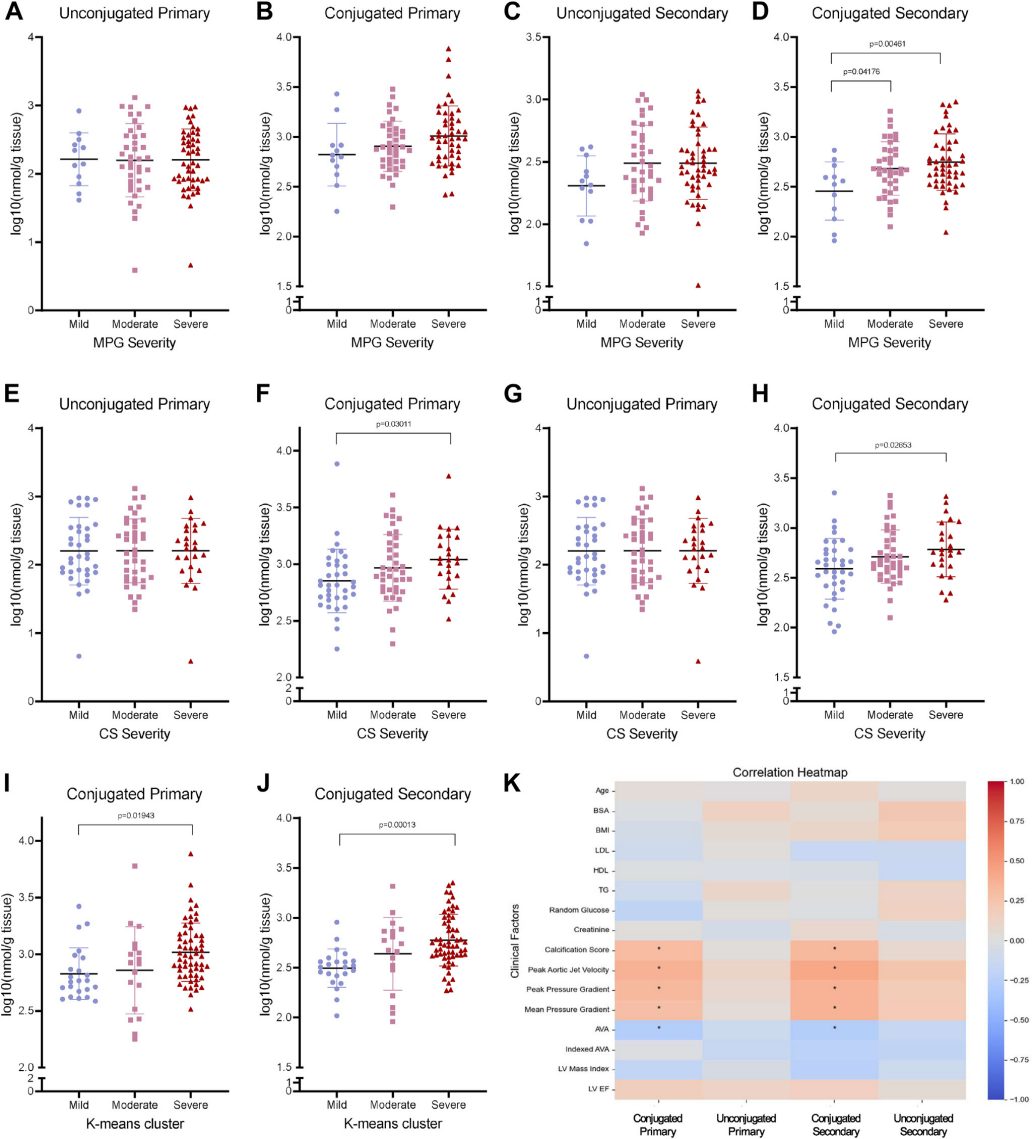
Analysis of conjugation and primary/secondary bile acids. A–D: Tissue levels of the grouped bile acids stratified by MPG groups. Only secondary-conjugated bile acids were increased in moderate and severe groups compared with mild. Although a trend of increasing conjugated primary bile acids with severity was present, it did not reach statistical significance. E–H: Tissue levels of the grouped bile acids stratified by calcification score severity. Conjugated primary and secondary bile acids were significantly higher in severe valves compared with mild valves. I–J: Conjugated primary and secondary bile acids were higher in severe K-means cluster valves compared with mild ones. K: A Pearson correlation heatmap of the bile acid groupings and clinical factors. Both primary and secondary bile acids had significant correlations with multiple echocardiographic parameters. *P* values <0.05 after multiple comparison adjustment are denoted with a “∗.”

A table of the conjugated and unconjugated primary and secondary bile acids that were analyzed can be found in supplemental Table S2. When analyzing total conjugated and unconjugated primary and secondary bile acids by MPG with one-way ANOVA, conjugated secondary bile acids were significantly elevated in the moderate and severe groups compared with mild group (Fig. 6D). There is also a trend of increasing conjugated primary bile acids with increasing MPG severity, although this did not reach statistical significance (Fig. 6B). When analyzing by calcification score, both conjugated primary and secondary bile acids are higher in severe compared with mild valve tissue, with no changes in the unconjugated bile acids (Fig. 6E–H). Similar to MPG and CS stratification, K-means clustering revealed higher conjugated primary and secondary bile acids in severe valves compared with mild valves (Fig. 6I, J). Furthermore, both primary- and secondary-conjugated bile acids significantly correlated with multiple echocardiographic parameters and had no correlations with baseline patient characteristics (Fig. 6K).

### Plasma bile acids

**Figure 7:**
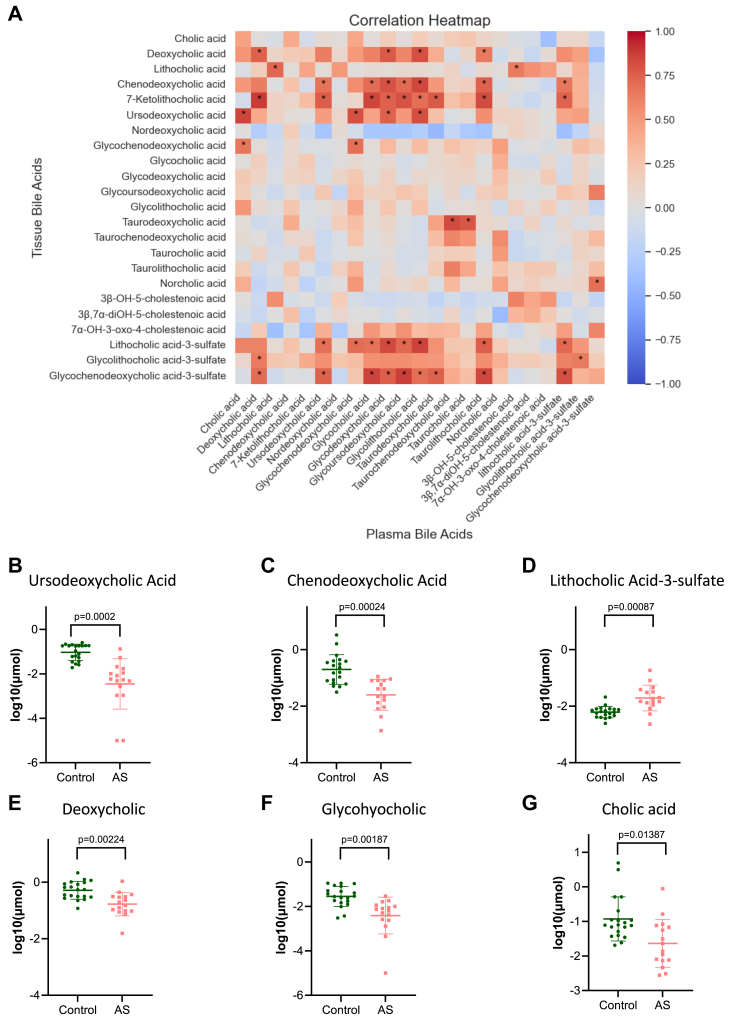
A: Pearson correlation heatmap of the tissue and plasma bile acids, with significant *P* values <0.05 after multiple comparison adjustment denoted with “∗.” B–G: Statistically significantly plasma bile acids between healthy controls and patients with moderate/severe aortic stenosis. Adjusted *P* values are as indicated on the graphs.

In a subcohort of 18 patients, we aimed to find the correlations between plasma and tissue bile acid profiles. Of the bile acids found to be significantly different by MPG and/or calcification score, plasma taurodeoxycholic and taurochenodeoxycholic acids were significantly correlated with their tissue levels (Fig. 7A). We then compared the plasma bile acids of healthy control plasma from a previously published cohort (15) (n = 20) to our moderately and severely diseased patients (n = 16, stratified by MPG). We found six plasma bile acids that were statistically different between AS patients and healthy controls (supplemental Fig. S1), including ursodeoxycholic acid, chenodeoxycholic acid, lithocholic acid-3-sulfate, deoxycholic acid, glycohyocholic acid, and cholic acid (Fig. 7B–G). Of these six bile acids, only lithocholic acid-3-sulfate was higher in AS patients compared with healthy controls.

## Discussion

**Figure 8:**
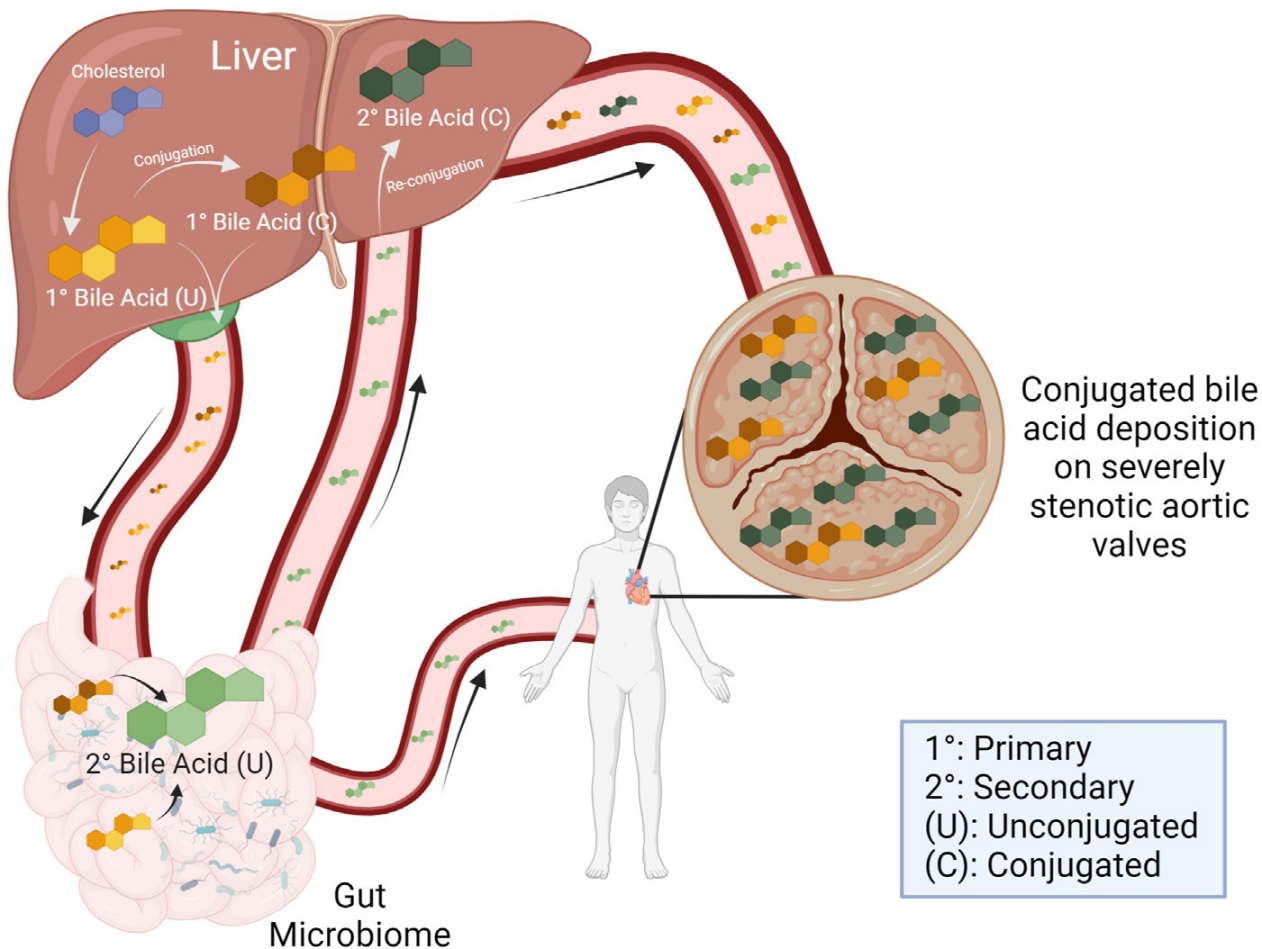
Primary bile acids are synthesized in the liver and can be conjugated there. The gut microbiome then deconjugates and/or dehydroxylates the bile acids to create secondary bile acids. These can then enter the circulation or be reconjugated in the liver. Conjugated bile acids are increasingly deposited on calcific aortic valves with increasing disease severity. Created with Biorender.com.

We present the first comprehensive analysis of bile acid alterations within human AV tissue in patients with severe CAVS. This finding is particularly significant given the current lack of medical therapies capable of slowing or halting the progression of CAVS. Moreover, this study has shown that a subset of bile acids—conjugated bile acids—are preferentially deposited on more severe valves, indicating there could be a physiologic process at play. As the global population continues to age, understanding the underlying mechanisms driving CAVS is becoming increasingly critical (Fig. 8).

In our previous untargeted analysis of AV tissue, bile acid metabolism was one of the key pathways identified. Building on these initial findings, we conducted a targeted, quantitative analysis of bile acids within human AV tissue. In this comprehensive targeted bile acid analysis, we screen for 79 bile acids, 23 of which were detectable in valvular tissue and 53 in plasma samples (supplemental Table S4). Stratifying patients by MPG and calcification severity, there was a significant increase in total bile acid levels with worsening gradient. Specifically, there were significant increases in norcholic acid, glycodeoxycholic acid, glycocholic acid, and taurodeoxycholic acid across all severe stratification groups. Notably, no significant differences in tissue bile acids were observed between moderate and severe groups using both stratification methods, suggesting these bile acids may contribute primarily to the early stages of CAVS progression.

Bile acids are traditionally recognized for their role in lipid digestion and cholesterol homeostasis with bile acids mediating cholesterol homeostasis by promoting its fecal excretion (18), but accumulating evidence highlights their broader impact on cardiovascular physiology. Bile acids interact with metabolic and signaling pathways, influencing vascular health and CVDs through inflammatory regulation, endothelial function, and calcium homeostasis (18, 19, 20, 21, 22). In the context of atherosclerosis, cholesterol accumulation in vascular walls leads to inflammatory recruitment and calcium deposition (23). Therefore, there may be potential for a similar mechanistic link in CAVS. However, in our study, we found very few or no correlations between valvular bile acids and circulating LDL and HDL, respectively, and the significant correlations were not related to valve disease severity (Fig. 4). This suggests circulating cholesterol elimination is likely not central to the mechanism of bile acid deposition in CAVS in our cohort.

On a cellular level, bile acids activate nuclear receptors that are known to be implicated in inflammation and calcium homeostasis, including the FXR and G-protein coupled receptor TGR5 (24, 25, 26). Specifically, both FXR and TGR5 are known to have anti-inflammatory properties in various organs, as well as stimulate vasodilation through increased nitric oxide (11, 22, 26). In vitro studies have shown that bile acids can stimulate endoplasmic reticulum stress by increasing intracellular calcium levels and reactive oxygen species (27). Bile acid receptor agonists have also shown to be potential therapeutic targets for chronic kidney disease and diabetes (11). The influence bile acids have on calcium and phosphate metabolism has implicated them in vascular calcification disorders (11). Certain bile acids including lithocholic acid and deoxycholic acids, both secondary bile acids, are involved in the osteoblastic differentiation of vascular smooth muscle cells and increased phosphate/calcium absorption (28, 29). Vascular calcification is linked to chronic inflammation and oxidative stress, both of which are influenced by bile acid metabolism. Studies have shown that while circulating levels of deoxycholic acid are correlated with increased coronary artery calcification and lower bone mineral density in chronic kidney disease patients (30, 31), it is not associated with atherosclerotic and heart failure events (32). Therefore, bile acids appear to not only promote direct cellular calcification but also can modulate the systemic calcium availability.

Primary bile acids are synthesized in the liver from cholesterol and can be conjugated with glycine or taurine. In the intestines, gut bacteria deconjugate and/or dehydroxylate these bile acids to form secondary bile acids, which may then be reconjugated in the liver (18, 33). Our study demonstrated that specifically conjugated primary and secondary bile acids are significantly lower in mildly diseased valves when classified by MPG, calcification score, and K-means clustering. The previous untargeted metabolomic study also showed increasing levels of tissue glycoursodeoxycholic acid, a secondary-conjugated bile acid, with MPG severity (9). Clinical data have already shown increased liver failure markers such as a high albumin-bilirubin score as independent risk factors for mortality in patients with severe AS (34). Furthermore, gut microbiome metabolites such as choline and trimethylamine N-oxide have clinically been shown to be associated with calcific AV stenosis (35, 36). Given the specificity of our observed trend and the important role of liver and gut microbiome in synthesizing conjugated bile acids, these results could suggest a potential link between these systems and valvular disease severity, which could be a new area of research interest. Bile acids are conjugated to increase their solubility and are less cytotoxic than their unconjugated counterparts (37). In fact, they have even recently been implicated as antihypertensive metabolites (38). Therefore, it is counterintuitive that we have found more conjugated bile acid deposition on more diseased valves, and these findings suggest more nuance and complexity in the regulation of these pathways. Given our study was observational, more studies are needed to determine if bile acids have a causative role in CAVS.

We observed a more prominent difference in conjugated secondary and conjugated primary bile acid deposition in the valves stratified by severity. The relationship between the digestive system, liver function, and valvular disease can be specifically explored in future studies to understand the mechanisms at play. As bile acid sequestrants already exist in clinical practice, it warrants further exploration on the role of bile acids in valvular disease to determine potential therapeutic benefits of pre-existing pharmacotherapies.

The key limitation of our study is the disproportionate skew toward moderate and severely diseased valves, as the majority of AV surgery are due to severe AV stenosis. But given the large number of our overall cohort, we stratified the patients based on MPG to create three groups. In comparing plasma from CAVS to healthy controls, none of the conjugated bile acids were significantly different, which could be a result of the small sample number and not age- or sex-matched controls. A larger sample size that is appropriately matched is likely needed to make meaningful conclusions on the role of circulating bile acids. Liver and gut parameters were also not measured in these patients, which are key components to the potential mechanistic aspect of these findings, and future studies are needed to understand the intricacies and roles of these multiorgan axes.

In conclusion, our study represents the largest and most comprehensive analysis of bile acids in human aortic valvular tissue from patients with CAVS. The composition of tissue bile is significantly altered with progression of disease. These findings underscore the potential for bile acid-based therapeutics in treating CAVS progression.

## Data availability

The datasets generated and analyzed during the current study are available from the corresponding author upon reasonable request. Patient-level data contain sensitive information and are not publicly available due to privacy and ethical restrictions.

## Supplemental data

This article contains supplemental data.

## Conflict of interest

The authors declare that they have no conflicts of interest with the contents of this article.
