# Supplementary material for: Conjugated bile acids are elevated in severe calcific aortic valve stenosis
Source: J Lipid Res. 2025 May 22;66(6):100830. doi: 10.1016/j.jlr.2025.100830 (PMC12214273; doi:10.1016/j.jlr.2025.100830)
Supplement: Supplemental data [file mmc1.pdf]

Supplementary Data:

Conjugated bile acids are elevated in severe calcific aortic valve stenosis

Hannah Zhang [1,2,3], Negar Atefi [1,4], Arun Surendran[1,2,3], Jun Han[4,5], David R. Goodlett[4,6], Davinder S. Jassal [2,8], Ashish Shah [2,7,8], and Amir Ravandi[1,2,7,8]

[1]Cardiovascular Lipidomics Laboratory, St. Boniface Hospital, Albrechtsen Research Centre,
[2]Department of Physiology and Pathophysiology, Rady Faculty of Health Sciences, University of Manitoba[3]Mass Spectrometry & Proteomics Core Facility, Rajiv Gandhi Centre for Biotechnology, Kerala, [4]Genome British Columbia Proteomics Centre, University of Victoria, Victoria, BC, Canada
[5]Division of Medical Sciences, University of Victoria, Victoria, BC, Canada
[6]Department of Biology and Microbiology, University of Victoria, Victoria, BC, Canada
[7]Precision Cardiovascular Medicine Group, St. Boniface Hospital Research.
[8]Section of Cardiology, Department of Internal Medicine, Rady Faculty of Health Sciences, University of Manitoba,

Corresponding author
Amir Ravandi MD, PhD, FRCPC
Cardiovascular Lipidomics Laboratory,
St. Boniface Hospital Albrechtsen Research Centre,
351 Tache Ave,
Winnipeg, MB Canada R2H 2A6
Phone.204-235-32315
Fax.204-235-0793
Email: amir.ravandi@umanitoba.ca

Table S1: Baseline characteristics of the patient population, stratified by k-means cluster.

Parameter	Total (n=100)	Cluster 1 (n=59) “Severe”	Cluster 2 (n=18) “Moderate”	Cluster 3 (n=23) “Mild”	P-value
Age, years (range)	69.2 ± 11.2 (33-89)	70.0 ± 10.4	68.9 ± 11.3	67.4 ± 13.4	0.65
Male, n	69	39 (66%)	13 (72%)	17 (74%)	0.75
Body surface area (m^2)	1.97 ± 0.22	2.00 ± 0.21	1.93 ± 0.19	1.97 ± 0.23	0.46
Body mass index (kg/m^2)	29.3 ± 5.6	30.4 ± 5.9	28.1 ± 4.6	28.9 ± 4.7	0.217
Hypertension, n	75	47 (80%)	12 (67%)	16 (70%)	0.42
**Smoking History, n**					
Current	10	9 (15%)	0 (0%)	1 (4%)	
Previous	48	26 (44%)	10 (56%)	12 (52%)	0.32
Never	42	24 (41%)	8 (44%)	10 (43%)	
**Medications, n**					
Anti-hypertensive	66	37 (63%)	12 (67%)	17 (74%)	0.63
ACE inhibitors*	30	18 (31%)	4 (22%)	8 (35%)	0.68
ASA*	28	17 (29%)	4 (22%)	7 (30%)	0.82
ARBs*	11	6 (10%)	3 (17%)	2 (9%)	0.68
Statins	61	38 (64%)	11 (61%)	12 (52%)	0.59
**Laboratory**					
LDL (mmol/L)*	2.63 ± 1.02	2.63 ± 1.07	2.89 ± 0.97	2.42 ± 0.89	0.34
HDL (mmol/L)*	1.36 ± 0.46	1.29 ± 0.43	1.49 ± 0.55	1.46 ± 0.44	0.14

TG (nmol/L)*	1.49 ± 0.82	1.45 ± 0.53	1.23 ± 0.40	1.79 ± 1.79	0.08
Random glucose (mmol/L)	7.20 ± 2.73	7.34 ± 2.78	6.61 ± 2.53	7.32 ± 2.79	0.60
Creatinine (µmol/L)	90.5 ± 38.1	88.2 ± 27.1	97.1 ± 44.8	91.0 ± 54.7	0.69
**Echocardiogram Data**					
Bicuspid valve, n	29	17 (29%)	5 (28%)	7 (30%)	0.98
Calcification Score	2.89 ± 1.02	3.14 ± 0.94	2.67 ± 1.14	2.43 ± 0.99	0.011
Peak aortic jet velocity (m/s)	4.03 ± 0.95	4.26 ± 0.80	3.79 ± 1.08	3.64 ± 1.04	0.013
Peak transvalvular gradient (mmHg)	68.8 ± 30.5	74.25 ± 30.22	64.0 ± 31.4	58.5 ± 28.3	0.082
Mean transvalvular gradient (mmHg)	41.6 ± 18.8	45.1 ± 18.5	37.5 ± 18.4	35.8 ± 18.5	0.076
Aortic valve area (cm^2)	0.95 ± 0.40	0.85 ± 0.23	1.15 ± 0.63	1.05 ± 0.47	0.007
Indexed aortic valve area (cm^2/m^2)	0.51 ± 0.35	0.43 ± 0.11	0.58 ± 0.30	0.68 ± 0.64	0.009
Left ventricular mass index (g/m^2)	127.4 ± 33.0	125.4 ± 29.3	121.2 ± 31.7	137.2 ± 41.4	0.23
Left ventricular ejection fraction (%)	56.4 ± 9.4	56.6 ± 9.5	57.8 ± 5.7	55.3 ± 11.5	0.71

P-values were obtained after subgroup comparison, with p-values<0.05 bolded. Values are mean ± SD, mean (range), numbers or % as applicable. Chi square test was used for categorical variables and one-way ANOVA (post-hoc Tukey) was used for continuous variables to assess for statistical significance between sample groups.

*Abbreviations: ACE, angiotensin-converting-enzyme; ASA, Acetylsalicylic acid; ARBs, angiotensin II receptor blockers; LDL, low-density lipoprotein; HDL, high-density lipoprotein; TG, triglyceride.

Table S2: List of Primary and Secondary Conjugated and Unconjugated Bile Acids

Unconjugated Primary Bile Acids	Conjugated Primary Bile Acids	Unconjugated Secondary Bile Acids	Conjugated Secondary Bile Acids
• Cholic acid<br>• Chenodeoxycholic acid	• Glycochenodeoxycholic acid<br>• Glycocholic acid<br>• Taurochenodeoxycholic acid<br>• Taurocholic acid	• Deoxycholic acid<br>• Lithocholic acid<br>• Ursodeoxycholic acid<br>• Norcholic acid<br>• Nordeoxycholic acid<br>• 7-Ketolithocholic acid	• Glycodeoxycholic acid<br>• Glycoursodeoxycholic acid<br>• Glycolithocholic acid<br>• Taurodeoxycholic acid<br>• Taurolithocholic acid'

Table S3. List of bile acids and Q1 to Q3 ion transitions for LC-MRM/MS measurements.

No.	Compound	Q1 m/z	Q3 m/z	RT (min)	Supplier
01	Taurodehydrocholic acid	508.2	80	4.5	Steraloids
02	Tauroursodeoxycholic acid-3-sulfate	578.2	80	4.8	IsoSciences
03	Tauro-ω-muricholic acid	514.3	80	5.3	Steraloids
04	Tauro-α-muricholic acid	514.3	79.9	5.7	Steraloids
05	Tauro-β-muricholic acid	514.3	79.9	5.9	Steraloids
06	Glycodehydrocholic acid	458.3	74	6.4	Steraloids
07	Glycoursodeoxycholic acid-3-sulfate	528.3	448.2	6.7	IsoSciences
08	Taurochenodeoxycholic acid-3-sulfate	578.2	80	6.9	IsoSciences
09	Taurodeoxycholic acid-3-sulfate	578.2	80	7.3	LGC Standard
10	Glycocholic acid-3-sulfate	544.3	464.2	7.4	Cayman Chem
11	Taurohyocholic acid	514.3	80	7.4	Steraloids
12	Glyco-ω-muricholic acid	464.3	74	7.5	Sigma-Aldrich
13	Glyco-α-muricholic acid	464.3	74	7.8	Steraloids
14	Glyco-β-muricholic acid	464.3	74	8	Cayman Chem
15	Tauroursodexycholic acid	498.3	80	8.5	Steraloids
16	Tauroallocholic acid	514.3	124.1	8.8	Steraloids
17	Taurocholic acid	514.3	124.1	9	Steraloids
18	Glycohyocholic acid	464.3	74	9.8	Steraloids
19	Ursocholic acid	407.3	407.3	10	LGC Standard
20	Glycochenodeoxycholic acid-3-sulfate	528.3	448.2	10.1	LGC Standard
21	Ursodeoxycholic acid-3-sulfate	471.2	97	10.1	LGC Standard
22	Taurolithocholic acid-3-sulfate	562.3	124	10.2	LGC Standard
23	DioxoLithocholic acid	403.3	403.3	10.3	Steraloids
24	Hyodeoxycholic acid-3-sulfate	471.2	97	10.5	Steraloids
25	Dehydrocholic acid	401.3	401.3	10.6	Steraloids
26	Glycodeoxycholic acid-3-sulfate	528.3	448.2	10.6	LGC Standard
27	Ursodeoxycholic acid-24-glucuronide	567.3	407.3	11.1	USBIO
28	Glycoallocholic acid	464.3	74	11.2	Steraloids
29	Glycoursodeoxycholic acid	448.3	74	11.2	Steraloids
30	Glycohyodeoxycholic acid	448.3	74	11.4	Steraloids
31	Glycocholic acid	464.3	74	11.5	Steraloids
32	ω-muricholic acid	407.3	407.3	11.6	Steraloids
33	α-Muricholic acid	407.3	407.3	12	Steraloids
34	Taurochenodexycholic acid	498.3	80	12.1	Steraloids
35	Norcholic acid	393.3	393.3	12.3	LGC Standard
36	Norursodeoxycholic acid	377.3	377.3	12.3	LGC Standard
37	7-Ketodeoxycholic acid	405.3	405.3	12.5	Steraloids
38	β-Muricholic acid	407.3	407.3	12.6	Steraloids
39	Taurodeoxycholic acid	498.3	80	12.8	Steraloids
40	GlycoLithocholic acid-3-sulfate	512.3	432.2	13.3	LGC Standard

41	12-Ketochenodeoxycholic acid	405.3	405.3	13.4	Steraloids
42	Deoxycholic acid-3-sulfate	471.2	97	13.5	LGC Standard
43	Chenodeoxycholic acid-3-sulfate	471.2	97	13.8	IsoSciences
44	λ-muricholic acid	407.3	407.3	13.8	Steraloids
45	Chenodeoxycholic acid-24-glucuronide	567.3	391.3	13.9	LGC Standard
46	Chenodeoxycholic acid-3-glucuronide	567.3	75	13.9	LGC Standard
47	Murocholic acid	391.3	391.3	14.1	Steraloids
48	Deoxycholic acid-3-glucuronide	567.3	75	14.2	LGC Standard
49	3-Oxocholic acid	405.3	405.3	14.4	Steraloids
50	Deoxycholic acid-24-glucuronide	567.3	391.3	14.5	USBIO
51	Allocholic acid	407.3	407.3	14.6	Steraloids
52	Cholic acid	407.3	407.3	14.9	Steraloids
53	Cholic acid-3-sulfate	487.2	97	14.9	IsoSciences
54	Glycochenodeoxycholic acid	448.3	74	15.1	Steraloids
55	Ursodeoxycholic acid	391.3	391.3	15.2	Steraloids
56	Hyodeoxycholic acid	391.3	391.3	15.4	Steraloids
57	Glycodeoxycholic acid	448.3	74	15.7	Steraloids
58	TauroLithocholic acid	482.3	80	15.8	Steraloids
59	Lithocholic acid-3-sulfate	455.2	97	16.3	Cayman Chem
60	6,7-DiketoLithocholic acid	403.3	403.3	16.6	Steraloids
61	Apocholic acid	389.3	389.3	16.6	Steraloids
62	7-KetoLithocholic acid	389.3	389.3	17	Steraloids
63	Nordeoxycholic acid	377.3	377.3	17.1	Steraloids
64	12-KetoLithocholic acid	389.3	389.3	17.2	Steraloids
65	Lithocholic acid-3-glucuronide	551.3	75	17.4	LGC Standard
66	Lithocholic acid-24-glucuronide	551.3	375.3	17.8	Cayman Chem
67	Chenodeoxycholic acid	391.3	391.3	18.5	Steraloids
68	Deoxycholic acid	391.3	391.3	19	Steraloids
69	GlycoLithocholic acid	432.3	74	19.1	Steraloids
70	3α,7α, 12α-Trihydroxy-5β-cholestanoic acid (THCA)	449.3	449.3	19.4	LGC Standard
71	3α,7α-Dihydroxycholestanoic acid (DHCA)	433.3	433.3	19.8	LGC Standard
72	3β,7α-diOH-5-cholestenoic acid	431.3	431.3	19.8	Sigma-Aldrich
73	7α-OH-3-oxo-4-cholestenoic acid	429.3	429.3	20.6	LGC Standard
74	AlloisoLithocholic acid	375.3	375.3	20.7	Steraloids
75	IsoLithocholic acid	375.3	375.3	21.1	Steraloids
76	Isodeoxycholic acid	391.3	391.3	21.3	LGC Standard
77	Lithocholic acid	375.3	375.3	22.4	Steraloids
78	DehydroLithocholic acid	373.3	373.3	22.9	Steraloids
79	3β-OH-5-cholestenoic acid	415.3	415.3	24.4	LGC Standard
IS	Chenodeoxycholic acid-d4	395.3	395.3	18.5	Steraloids
IS	Cholic acid-d4	411.3	411.3	18.5	Steraloids
IS	Deoxycholic acid-d4	395.3	395.3	19	Steraloids

IS	Glycochenodeoxycholic acid-d4	452.3	74	15.1	Steraloids
IS	Glycocholic acid-d4	456.3	74	15.1	Steraloids
IS	Glycodeoxycholic acid-d4	452.4	74	15.7	CDN Isotopes
IS	Glycolithocholic acid-d4	436.3	74	19.1	CDN Isotopes
IS	Glycoursodeoxycholic acid-d4	452,3	74	11.2	IsoSciences
IS	Lithocholic acid-d4	379.3	379.3	22.4	Steraloids
IS	Taurochenodeoxycholic acid-d4	502.3	80	12.1	IsoSciences
IS	Taurocholic acid-d4	411.3	411.3	14.9	IsoSciences
IS	Taurodeoxycholic acid-d4	502.3	80	12.8	IsoSciences
IS	Taurolithocholic acid-d4	486.3	80	15.8	IsoSciences
IS	Ursodeoxycholic acid-d4	395.3	395.3	15.2	CDN Isotopes

Table S4. List of detectable bile acids in tissue and plasma.

**Tissue Bile Acids**	**Plasma Bile Acids**
• Cholic acid<br>• Deoxycholic acid<br>• Lithocholic acid<br>• Chenodeoxycholic acid<br>• 7-Ketolithocholic acid<br>• Ursodeoxycholic acid<br>• Nordeoxycholic acid<br>• Glycochenodeoxycholic acid<br>• Glycocholic acid<br>• Glycodeoxycholic acid<br>• Glycoursodeoxycholic acid<br>• Glycolithocholic acid<br>• Taurodeoxycholic acid<br>• Taurochenodexycholic acid<br>• Taurocholic acid<br>• Taurolithocholic acid<br>• Norcholic acid<br>• 3β-OH-5-cholestenoic acid<br>• 3β,7α-diOH-5-cholestenoic acid<br>• 7α-OH-3-oxo-4-cholestenoic acid<br>• Lithocholic acid-3-sulfate<br>• Glycolithocholic acid-3-sulfate<br>• Glycochenodeoxycholic acid-3-sulfate	• Cholic acid<br>• Deoxycholic acid<br>• Lithocholic acid<br>• Allocholic acid<br>• Chenodeoxycholic acid<br>• Dehydrocholic acid<br>• Dehydrolithocholic acid<br>• 7-Ketolithocholic acid<br>• Murocholic acid<br>• Ursodeoxycholic acid<br>• Isolithocholic acid<br>• Nordeoxycholic acid<br>• β-Muricholic acid<br>• Glycochenodeoxycholic acid<br>• Glycocholic acid<br>• D_Glycoallocholic acid<br>• D_Glyco-a-muricholic acid<br>• D_Glyco-w-muricholic acid<br>• Glycodeoxycholic acid<br>• Glycoursodeoxycholic acid<br>• Glycolithocholic acid<br>• Glycohyocholic acid<br>• Taurodeoxycholic acid<br>• Taurochenodexycholic acid<br>• Taurocholic acid<br>• Taurolithocholic acid<br>• Tauro-α-muricholic acid<br>• Tauro-β-muricholic acid<br>• Tauro-ω-muricholic acid<br>• Norursodeoxycholic acid<br>• Norcholic acid<br>• 3β-OH-5-cholestenoic acid<br>• 3β,7α-diOH-5-cholestenoic acid<br>• 7^{α}-OH-3-oxo-4-cholestenoic acid<br>• DHCA<br>• THCA<br>• Chenodeoxycholic acid-3-glucuronide<br>• Ursodeoxycholic acid-3-glucuronide<br>• Ursodeoxycholic acid-24-glucuronide<br>• Lithocholic acid-3-sulfate<br>• Deoxycholic acid-3-sulfate<br>• D_deoxycholic acid-3-sulfate<br>• D_ursodeoxycholic acid-3-sulfate<br>• D_β-muricholic acid-3-sulfate<br>• Glycolithocholic acid-3-sulfate<br>• Glycodeoxycholic acid-3-sulfate<br>• Glycochenodeoxycholic acid-3-sulfate<br>• Glycoursodeoxycholic acid-3-sulfate<br>• D_Glycohyodeoxycholic acid-3-sulfate<br>• Taurolithocholic acid-3-sulfate<br>• Taurodeoxycholic acid-3-sulfate

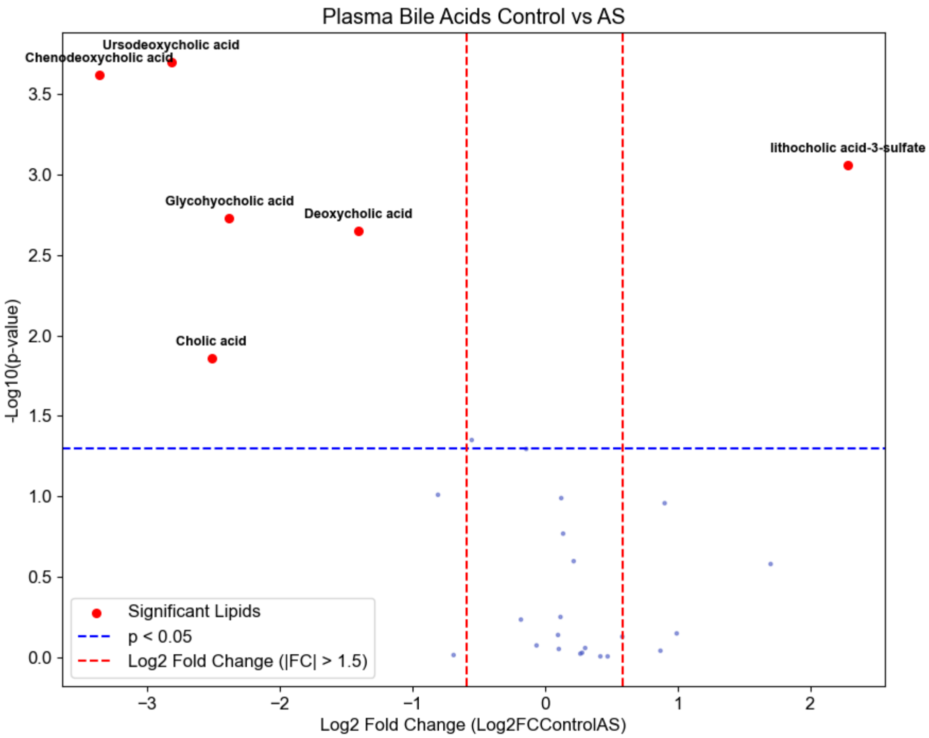

Figure S1. Volcano plot of significantly different plasma bile acids between 20 healthy controls and 16 moderate or severe aortic stenosis (AS) patients. Statistically different bile acids are red as indicated.
